# Amperometric Inkjet-Printed Thyroxine Sensor Based on Customized Graphene and Tunned Cyclodextrins as the Preconcentration Element

**DOI:** 10.3390/nano14050403

**Published:** 2024-02-22

**Authors:** María Jesús Ortiz-Aguayo, Franc Paré, Gemma Gabriel, Mireia Baeza

**Affiliations:** 1 Department of Chemistry, Faculty of Science, Edifici C-Nord, Universitat Autònoma de Barcelona, Carrer dels Tillers, 08193 Bellaterra, Spain; mortiz@icmab.es (M.J.O.-A.); franc.pare@uab.cat (F.P.); 2 GENOCOV Research Group, Universitat Autònoma de Barcelona, 08193 Bellaterra, Spain; 3 Instituto de Microelectrónica de Barcelona, IMB-CNM (CSIC), Esfera UAB, Campus Universitat Autònoma de Barcelona, 08193 Bellaterra, Spain; gemma.gabriel@imb-cnm.csic.es; 4 Centro de Investigación Biomédica en Red de Bioingeniería, Biomateriales y Nanomedicina (CIBER-BBN), 28029 Madrid, Spain

**Keywords:** thyroid hormones, electrochemical (bio)sensor, graphene, β (beta)-cyclodextrin, γ (gamma)-cyclodextrin

## Abstract

The determination of thyroid hormones has practical clinical significance for the diagnosis of hyperthyroidism and hypothyroidism diseases. Considering this aspect, a wide range of analytical methods for the detection of analytes, including immunoassay, chemiluminescence, mass spectroscopy and high-performance liquid chromatography, among others, has been developed. This type of analysis provides feasible results. Nevertheless, it requires qualified staff, special facilities and is time-consuming. For this reason, this paper relies on the fabrication of an electrochemical device developed with inkjet printing technology for the free detection of Thyroxine (T4). To manufacture our electrochemical device, several aspects were considered from the use of materials that amplify electrical signals, to finding a supramolecular scaffold that possess affinity towards the target analyte and the need of preconcentrating the analyte on the electrode’s surface. For this task, printed devices were modified with a hybrid nanomaterial consisting of reduced graphene oxide (rGO) tuned with Au nanoparticles (Au–NPs) and an entrapment agent and different thiolated cyclodextrins (x–CD-SH) as carrying agents. Analytes were preconcentrated via supramolecular chemistry due to the formation of an inclusion complex between the cyclodextrin and hormones. Morphological and electrochemical characterization of the final device was carried out to ensure the proper workability of the electrode, achieving excellent response, sensitivity and limit of detection (LOD).

## 1. Introduction

Thyroxine (T4) is an important hormone that is considered to be an iodoamino acid derivative of thyronine (T3), and they are both released by the thyroid gland. Thyrotropin (TSH) is a pituitary hormone that is secreted by the pituitary gland as is shown in Figure S1. The gland is regulated by thyrotropin-releasing hormone (TRH), which is produced in the hypothalamus. The presence of TSH activates the thyroid gland, located behind the larynx, which regulates the release of T4 and T3 [1].

The determination of thyroid hormones has practical clinical meaning for the diagnosis of hyperthyroidism and hypothyroidism diseases. Both illnesses are produced by a hormonal imbalance, increasing or decreasing the optimum segregation levels of the hormones, which range from 0.8 to 1.33 ng/dL for T4 (free); 60 to 180 ng/dL for T3 (total); and 0.63 to 4.19 µIU/mL for TSH [2]. Several analytical methods for the detection of the studied analytes have been developed, including chemiluminescence [3], mass spectroscopy [4], high-performance liquid chromatography [5], and immunoassay [6]. Immunoassays, specifically enzyme-linked immunoassay (ELISA), are considered one of the most sensitive methods for hormone quantification in blood, providing analysis with feasible results [7]. Nevertheless, most of these methods require qualified staff, special facilities and are time-consuming. To solve these disadvantages, electrochemistry has emerged as an alternative strategy for the development of updated analytical methodologies to be implemented in point-of-care (POC) devices. Electrochemical techniques can serve as useful tools for the detection of not just the analyte of interest, but also some of the precursor species such as phenylalanine [8] or tyrosine [9,10]. Whilst they are chemically similar to the target of interest, they are not correlated directly with the disorders mentioned.

Several works report the study of T4′s electrochemical response by employing different types of sensing platforms that can help to improve simple and reliable detection tools to effectively monitor levels during treatments in a miniaturized way [11,12]. There is a specific focus on platforms that are grounded using carbon-based materials and screen-printed electrodes [13,14,15], as they are great candidates for the fabrication of miniaturized (bio)sensors with high sensitivity and ease of(bio)functionalization. They can help to improve simple and reliable detection tools to effectively monitor T4 levels during treatment.

Moreover, these materials are suitable to be used with polymer matrices to form composites, thereby enhancing their mechanical and electrochemical properties [16]. At this juncture, the relevance of investigating graphene-based composite sensors is considered. Graphene-based 2D hybrid materials present improvements in their electrochemical properties compared to conventional carbon materials, such as graphite or black carbon, which are mainly attributed to the presence of more sp^2^-like planes and edge defects in graphene making the material more electrochemically active. In addition, thanks to their malleability, conductive elements such as gold nanoparticles (Au–NPs@rGO) are employed as modifiers by promoting electron transfer by catalyzing the redox processes of the electroactive species and providing an ideal atmosphere for (bio)molecule immobilization [17,18,19]. As was mentioned before, carbon-based materials could provide enhanced properties in electrochemical sensors. Nevertheless, supramolecular systems can be integrated into sensor designs to give enhanced sensitivity and improve the limit of detection for the target analyte. Cyclodextrins (γ/β–CD) could act as preconcentration elements by building inclusion complexes through establishing interaction with analytes, specifically T4, through their hydrophobic cavities [20,21]. Finally, an important aspect to be considered is the miniaturization and manufacturing of electrochemical systems [22]. Recently developed technologies such as printed electronics (PE) have allowed the fabrication/miniaturization of electronic devices, which offer great advantages compared to microelectronics’ traditional construction processes due to their versatility, low-cost production and the possibility to generate flexible electronics. Inkjet printing (IJP) is a non-contact digital printing technique that involves the injection of small drops of ink (1 or 10 pL) in a precise place, giving rise to the manufacturing of narrow conductive tracks and very thin layers (<0.3 μm). This fact reduces the device’s size and cost, as well as the amount of waste material and manufacturing time, producing high-resolution patterns at the same time [23]. For that matter, a system based on Au–NPs@rGO with attached thiolated CD (γ/β–CD-SH), manufactured by means of IJP technology, is a novel and great alternative for the construction of an amperometric biosensor for thyroxine detection.

In this work, device development is achieved by first optimizing the inkjet printing protocol, which allows the robust and stable fabrication of gold working electrodes. Following that, different approaches were tested in order to successfully modify the gold’s surface with the carbon-based material filled with gold nanoparticles and the preconcentration elements. Finally, the sensor was electrochemically characterized, and the linear concentration range of the thyroxine was determined. In addition, stability and interference studies were carried out to ensure that the signal obtained corresponded entirely to the formation of the host–guest complex.

## 2. Materials and Methods

For the development of the microelectrodes, a silver nanoparticle ink (Dupont-PE140 from Dupont, Wilmington, DE, USA), gold colloidal ink (DryCure Au–JB 1010B from Colloidal Ink Co., Ltd., Soja, Okajama, Japan) and SU-8 dielectric photoresist (2002 from Kayaku Advanced Materials, Westborough, MA, USA) were employed. A polyethylene terephthalate (PET) of 125 μm (Melinex ST 504 Dupont Teijin Films, Richmond, VA, USA) was chosen as the substrate for device printing.

Graphene oxide (GO) was synthesized from flaked graphite (Sigma-Aldrich, St. Louis, MO, USA) using Hummers’ method. Reduced graphene oxide (rGO) was obtained through the reduction of GO using ascorbic acid [24]. Entrapment elements such as per-6-thio-β-cyclodextrin (β–CD-SH, 95%) and γ-cyclodextrin (γ–CD, 97%) were provided by AraChem (Tilburg, The Netherlands) and Biosynth Carbosynth (Bratislava, Slovakia), respectively. Note that γ–CD was thiolated synthetically [25], obtaining per-6-thio-γ-cyclodextrin (γ–CD-SH) as the desired reagent in situ.

Chitosan, polyethylene glycol (PEG, M_w_ = 4000), lignin and polylactic acid (PLA, M_w_ = 60,000) were purchased from Sigma-Aldrich (St. Louis, MO, USA).

All solutions were prepared using deionized water from a Milli-Q system (Millipore, Billerica, MA, USA) with a resistivity value of 18.2 MΩ·cm. Potassium ferricyanide and potassium ferrocyanide (K_3_[Fe(CN)_6_] and K_4_[Fe(CN)_6_], 99.8%), potassium chloride (KCl, 99.5%), sodium chloride (NaCl, 99.5%) phosphate-buffered saline tablets (PBS), ethanol CH_3_CH_2_OH > 99.5%), ascorbic acid (C_6_H_8_O_6_, 99.5%), Levo-Thyroxine (T4 or 3,5,3′,5′-tetraiodothyroxin > 98%), uric acid (C_5_H_4_N_4_O_3_, 99.5%) and DL-lactic acid (CH_3_CH(OH)COOH, 85%) were all purchased from Sigma-Aldrich (St. Louis, MO, USA).

A stock solution of 5 mM T4 was prepared by dissolving it in a 0.1 M HCl solution and storing it in the dark at 4 °C. Standard solutions were prepared by diluting the stock solution.

The interference experiments were carried out per triplicate (n = 3) by analyzing the interfering species in blood serum simulated composition in a PBS solution containing an ionic background of 0.1 M KCl and a residual background of 10 nM T4. The concentrations employed for NaCl, KCl, uric acid and lactic acid were 1.3, 1.5, and 3.5 mmol/L, respectively.

### 2.1. Inkjet-Printed Electrode Fabrication

A drop-on-demand Dimatix Material Printer (DMP 2831, FUJIFILM-Dimatix, Inc., Santa Clara, CA, USA) was used to print the different materials. The cartridges and printheads were also acquired from FUJIFILM-Dimatix, with a nominal drop of 10 pL.

**Figure 1:**
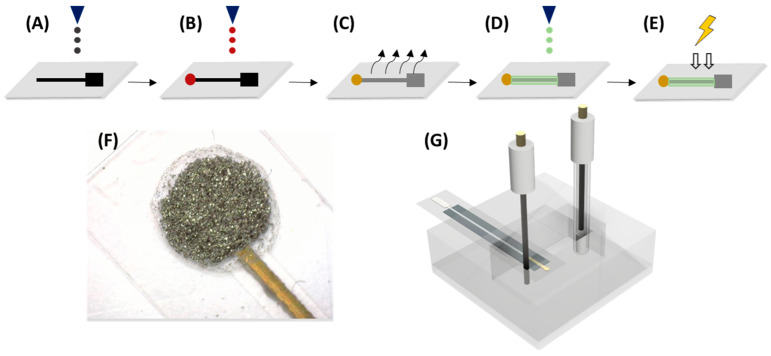
Overview of manufacturing procedure for sensor fabrication. Inkjet-printing (IJP) steps for sensor fabrication. (**A**) Ag tracks and pads; (**B**) Au electrode; (**C**) thermal sintering; (**D**) electrode passivation using electrical insulator; (**E**) UV curing.; (**F**) Is a photograph of the resulting microelectrode after the drop-casting process has been applied, whilst (**G**) is a schematic of the cell used for measurements.

Figure 1 shows the overall printing procedure, which is slightly modified from Moya et al. [26]. Firstly, two layers of Ag ink were printed, using a drop-spacing of 30 µm, to obtain the connection track and pad of the electrode. Secondly, the Au microelectrode was printed with a 20 µm drop spacing. Then, the drying step was completed on a hot plate, where the substrate was submitted to 80 °C for 15–20 min. Subsequently, the sintering procedure was carried out for 1 h at 140 °C. After the printing of the metallic materials, a layer of polymeric SU-8 ink was printed utilizing a drop-spacing of 15 µm, and it was cured by UV treatment for 15 s.

### 2.2. The Electrodes’ Surface Functionalization

10 mL of a solution containing 1 mg·mL^−1^ of graphene-based materials (see Supplementary Materials) was dispersed in Milli-Q water by sonicating for 1 h.

Afterwards, 100 μL of different polymer-tested 1% solution was added to the mixture to create the composite dispersion, which was directly drop-casted onto the electrodes. The employed volume for the drop casting mixture was 5 μL, split into 5 drops that were added in 5 min intervals. The electrodes were modified on a hot plate at 50 °C. In all polymer dispersions, the rGO/polymer ratio was kept constant. Once all the layers had been drop-casted, the electrodes were dried at 40 °C for 1 h.

To functionalize Au–NPs@rGO with an entrapment element, β–CD-SH or γ–CD-SH, an aqueous Au–NPs@rGO suspension was added to a 500 mL round-bottomed flask and set to stir at room temperature for 30 min. Then, 250 mL of an aqueous (9:1 water/NaOH, *v*/*v*) 2.0 mM dispersion of thiolated x–CD was added. The solution was left to stir overnight. Afterwards, the product was centrifuged at 3500 rpm for 10 min and washed several times with Milli-Q water and NaOH 10 mM, to remove the x–CD-SH that had not attached. The resultant product (x–CD-S/Au–NPs@rGO) was dried overnight at 40 °C.

### 2.3. Characterization of Hybrid Nanomaterials

A high-resolution transmission electron microscope (HR–TEM, JEM-1400, Jeol, Tokyo, Japan) and energy dispersive X-ray spectroscopy (EDS) were used to obtain images of the hybrid nanomaterial. In addition, an optical microscope (DM 4000M from Leica, Wetzlar, Germany) and scanning electron microscope (SEM, Auriga-40 from Carl Zeiss, Oberkochen, Germany) were employed to obtain microscopic images of the electrode surface containing carbon materials and its corresponding customization with nanoparticles. The thermogravimetric analysis (TGA, STA 449 F1 Jupiter^®^ from Netzsch, Sant Cugat del Vallès, Spain) technique was carried to quantify the total metal content in the nanostructured carbon materials. Approximately 1 mg of the sample was heated to 1000 °C at 10 °C/min using air flow. The mass of the sample was continuously measured as a function of temperature and the rate of weight loss (d.t.g.) was automatically recorded.

### 2.4. Electrochemical Characterization and Sensor Performance

Electrochemical measurements were performed using a potentiostat/galvanostat (Metrohm PGSTAT204, Utrech, The Netherlands) and the NOVA (v. 2.1.4) software package. A three-electrode configuration cell was used for electrochemical measurements, employing a micro reference electrode Ag/AgCl with 1.0 M KCl as an internal reference solution and a Pt-wire counter-electrode, both from Italsens (PalmSens BV, Houten, The Netherlands). The different inkjet-printed electrodes modified with nanocomposites based on carbon materials were used as working electrodes (WEs).

To ensure optimal response, an IJP bare Au electrode (WE) requires a prior activation before its functionalization, which can be induced by applying potential pulses ranging from 0 to −2 V vs. Ag/AgCl in a PBS solution. Sequentially, in order to verify if an optimal activation response had been achieved, cyclic voltammetry (CV) measurements were performed in the redox pair K_3_[Fe(CN)_6_]/K_4_[Fe(CN)_6_] 10 mM in KCl 0.1 M. These measurements were also used to calculate the charge transfer resistance and electroactive area of the modified electrodes with different materials. The electroanalytical response of the sensors was studied using the chronoamperometry technique. Before amperometric experiments, CV measurements were taken to determine the working potential (E_w_) of thyroxine oxidation for electroanalytical detection. For electrode calibration, the current intensities for the anodic curves were registered after the addition of the T4 stock solution of 1.0 µM that was dissolved in PBS solution containing an ionic background of 0.1 M KCl. After each calibration, the sensor was immersed in the buffer (PBS, 0.1 M KCl) for 10 min to remove the unoxidized T4 retained in the cyclodextrin. Finally, the chronoamperometric responses of some of the interferents of the developed sensor were monitored by analyzing the potential interfering species by dissolving the simulated blood serum composition in a PBS solution containing a residual background of thyroxine. In separate solutions, all containing 10 nM T4 as a signal baseline, 1.3 mM of NaCl, 1.5 mM of KCl, 3.5 mM of uric acid and 3.5 mM of lactic acid were measured in 0.1 M NaOH 1:9 in EtOH. A solution without interferents and 10 nM of T4 was also measured, and its signal was subtracted from those of the previous solutions. Then, all signals were normalized from the no-interferent’s current.

## 3. Results and Discussion

The characterization and study of the stability of the drop-casted dispersions containing rGO over the Au electrodes were initially performed. The main objective of this was to achieve an optimal composition ratio that would produce a robust, long-lasting and well-adhered dispersion. To achieve this goal, several compositions based on biodegradable, natural and synthetic polymers such as lignin, chitosan, polyethylene glycol (PEG) and polylactic acid (PLA) were tested. As seen in Figure S2A, after drop-casting, all of the electrodes were evenly distributed on the Au microelectrode’s surface. Moreover, the adhesion was not good, as after 20 min of being immersed in water, there was a loss of conductive material for lignin and PEG. In addition, the CV measurements of the Au electrode and the electrodes modified with chitosan and PLA composites were evaluated. Figure S2B shows that a signal increase (I_p_) was obtained respective to the bare electrode. The chitosan also had a better electrochemical performance than the PLA. Based on the results of the tested composites, the conclusion obtained is that chitosan is the optimum polymer for forming the drop-casted composite dispersion, showing proper stability and enhancing the electrochemical response of the bare electrodes.

The physical characterization of the graphene-based materials was performed to study their feasibility. HR–TEM images were taken for the different graphene-based nanomaterials (Figure S3). Figure S3A demonstrates the successful synthesis of rGO from bulk graphite. The picture presented in Figure S3B depicts an image of the homogeneous deposition of gold nanoparticles upon the graphene’s surface, resulting in a Au–NPs@rGO hybrid nanomaterial with an average Au particle of (4.4 ± 0.4) nm (Figure S3E). Figure S3C,D show the resulting hybrid nanomaterial after attaching the entrapment element (X–CD-SH) to the AuNPs@rGO sheets. Furthermore, a qualitative demonstration of the presence of different nanomaterials (Au–NPs@rGO and x-CD-S/Au–NPs@rGO) was obtained by EDS (Figure S4), which confirms the presence of gold (Figure S4A) and a sulfur peak (Figure S4B,C) by the incorporation of thiolate cyclodextrin, verifying the successful attachment of the carrying agent on the Au–NPs surface in both cases.

**Figure 2:**
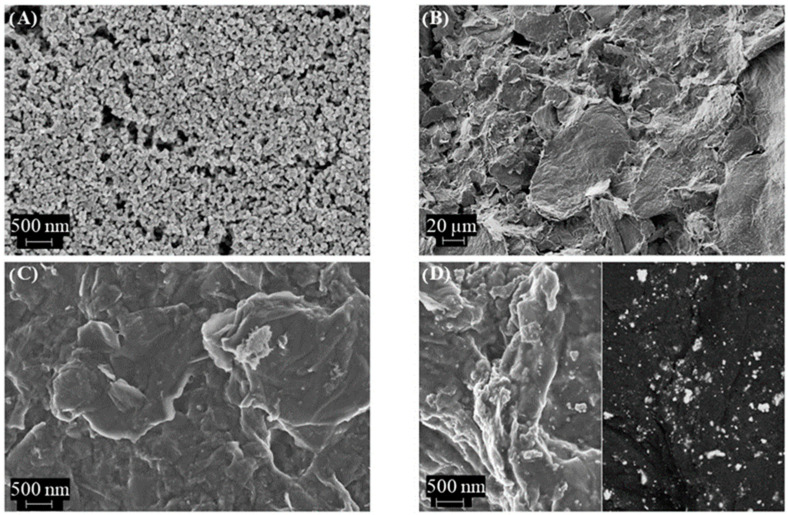
SEM images of (**A**) bare Au inkjet-printed electrode, (**B**,**C**) rGO: chitosan electrode at different scale and (**D**) Au–NPs@rGO: chitosan electrode using secondary electrons (**left**) and back-scattered electrons (**right**) at the same scale.

In addition, the SEM images show how the nanoparticle-based topography of the Au microelectrode was modified after composite deposition (Figure 2). In such cases, well-exfoliated rGO sheets can be observed at different magnification (Figure 2B,C), and the observation of gold nanoparticles (Au–NPs@rGO) as brilliant dots dispersed uniformly in the carbon material is considered (Figure 2D).

Further characterization was performed through thermal analysis. Thermogravimetry (Figure S5) was used to quantify the composition of the drop-casted graphene-like materials, both with and without cyclodextrins. With this data, an approximate calculation of the amount of β-cyclodextrin and γ-cyclodextrin on the surface of the sensor was performed (Supplementary Materials) [27].

The following electrodes were fabricated to compare their electrochemical performance: the bare Au electrode and the modified ones, rGO, Au–NPs@rGO, β–CD-S/Au–NPs@rGO and γ–CD-S/Au–NPs@rGO. CV measurements were taken under the same experimental conditions, in the presence of the benchmark [Fe(CN)_6_]^3−/4−^ redox couple (Figure S6). Table S1 shows that the bare Au electrode reports an increase in the current intensity and electroactive area (A) values, as well as the decrease in the charge–transfer resistance (R_ct_) when the Au surface’s electrode is modified with rGO. This fact demonstrates that rGO acts as a proper transductor, as it increases the roughness of the bare Au electrode and, therefore, its electroactive area. In addition, when the surface electrode is modified with Au–NPs@rGO, an increase in the peak height is observed. This enhancement can be attributed to the electrocatalytic effect of functional metal-nanoparticles on electrochemical systems. However, when the preconcentration agent (β–CD-S/γ–CD-S) is attached to Au-NPs, the current height value (I_p_) significantly decreases. This is mainly caused by the steric hindrance of these molecules on the electrode’s surface. Moreover, the I_p_, A and R_ct_ are higher than those obtained for the Au bare electrode. Despite this fact, the bare electrode modified with the 1:10 mixture of the chitosan:β–CD-S/Au–NPs@rGO nanocomposite still maintains an excellent electrochemical performance; therefore, its function as a carrying element does not affect the effective electrochemical behavior of the electrode.

**Figure 3:**
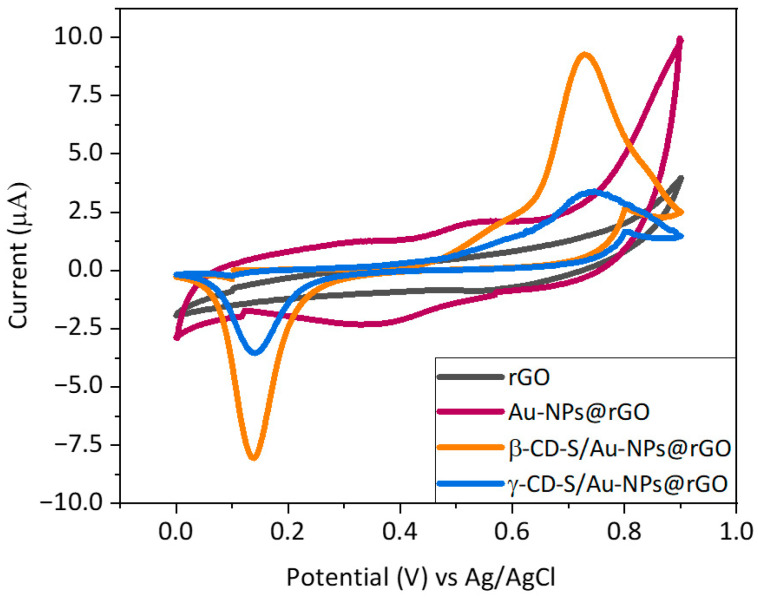
CV measurements of the different IJP Au electrodes modified with graphene and chitosan nanocomposites studied under the presence of 1 μM of T4 in a 0.1 M NaOH 1:9 in EtOH solution on a PBS solution with 0.1 M KCl. Scan rate of 10 mV·s^−1^.

Furthermore, electrochemical behavior was evaluated by means of CV, and responses to the analyte T4 were compared (Figure 3). From the graph, there is a notable pair of anodic and cathodic peaks (Ea_1_/Ec_1_) when using the IJP Au–NPs@rGO sensor of around +0.55 V vs. Ag/AgCl and +0.35 V vs. Ag/AgCl, respectively, which are believed to be due to the Au-NPs redox activity. On the other hand, a well-defined oxidation (Ea_2_) and reduction (Ec_2_) peak appeared around +0.70 V and + 0.19 V vs. Ag/AgCl for the electrooxidation and reduction, respectively, of thyroxine on the modified electrodes surface. Eventually, the relevance to introduce x-CD-S as a preconcentration agent in the device was also verified in terms of T4-oxidation detection. By changing the scanning speed, the current intensity derived from the electrochemical reaction of a species in a solution must linearly increase with it. As this was the observed behavior for the oxidation signal of +0.75 V vs. Ag/AgCl in a solution containing T4, it was concluded that it is due to the oxidation of T4 (Figure S7). As expected, the Au bare electrode modified only with rGO did not present electrochemical activity in the presence of thyroxine. Again, these results confirm the successful functionalization of the graphene sheets. In addition, the introduction of the carrying agent to the microelectrode considerably increased the current signal of the T4 oxidation (Ea_2_) by the β/γ–CD-S/Au–NPs@rGO sensors. This rise in the oxidation peak current at 0.75 V vs. Ag/AgCl indicates that there is a strong supramolecular interaction between both entrapment agents and the analyte. However, these interactions are more favorable for β–CD than for γ–CD, resulting in a higher peak intensity for β–CD.

**Figure 4:**
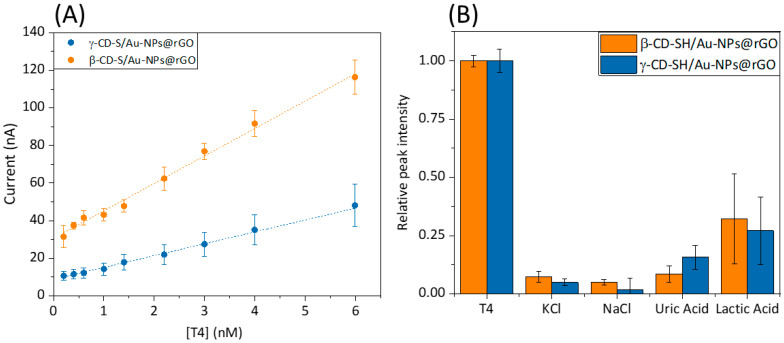
(**A**) Calibration curves from the representation of I_p_^a^ vs. [T4] for n = 3 were obtained by chronoamperometric measures in a 0.1 M NaOH 1:9 in EtOH solution on a PBS solution with 0.1 M KCl. E_w_: +0.75 V vs. Ag/AgCl. Error bars correspond to one standard deviation. (**B**) Interference study using different species in the developed T4 detection method, simulating each concentration at plasma levels ([NaCl] = 1.3 mmol/L, [KCl] = 1.5 mmol/L and [uric acid] and [lactic acid] = 3.5 mmol/L) and [T4] fixed at 10 nM in a 0.1 M NaOH 1:9 in EtOH solution. E_w_ = + 0.75 V vs. Ag/AgCl.

Consequently, electroanalytical characterization was conducted (Table 1). An amperometric calibration curve for both of the β/γ–CD-S/Au–NPs@rGO-modified sensors in PBS solution with subsequent additions of a 1.0 µM T4 aliquots was calculated. Figure 4A shows the calibration curve of β–CD-S/Au–NPs@rGO and γ–CD-S/Au–NPs@rGO. Calibration curves were performed by chronoamperometry at +0.75 V vs. Ag/AgCl (Figure S8). The current intensities, once they had stabilized, were taken as the sensor’s signal. Both calibration curves show a straight line with a concentration range from 0.1 nM to 6 nM for T4 (Figure S9). This weaker binding affinity of γ–CD for T4 compared to β–CD likely explains the lower ability of γ–CD to preconcentrate T4 on the modified microelectrode surface. As a result, fewer bound T4 molecules lead to fewer electron transfers, and a lower oxidation current measured for γ–CD compared to β–CD was obtained. The limit of detection (LOD) of the nanocomposite microsensors modified with the hybrid nanomaterials as preconcentration elements was calculated using S/N = 3 criterium [28]. The limit of quantification (LOQ) of the microsensors was calculated as S/N = 10 criterium. Specifically, better LOD was obtained for β–CD (0.25 nM) compared to γ–CD (0.89 nM). Lower LODs values ensure an optimal S/N ratio and T4 preconcentration on the surface of the modified microsensor. Levels that were detectable by the sensing platform are closer to the diagnostic levels of the disease (pM concentration range).

Regarding further application of the developed sensor with real samples, it should be considered that human blood serum contains many more bio components, like salts, amino acids, carbohydrates and lipids, among others. Those elements can interfere with T4 detection by lowering the sensitivity and increasing the LOD of both biosensors. Therefore, the amperometric response of some co-existing components of a different nature in blood serum were examined in the recognition system. From Figure 4B, it is quite evident that salts have negligible interference, due to the weak inclusion complex formed between these analytes and x-CD. To study these effects, currents were recorded by chronoamperometry at +0.75 V vs. Ag/AgCl in a 0.1 M NaOH 1:9 in EtOH solution on a PBS solution with 0.1 M KCl ionic buffer, one interferent at a time. For easier data analysis, the signals were normalized corresponding to the T4 response. Referring to uric and lactic acid, a signal enhancement is obtained, which can be explained by the similarity of the interactions of the recognition agent with lactic acid in comparison to those of the analyte. Nevertheless, the ratio of intensities confirms that the sensors detect T4 efficiently in the concentration range without signal hampering from the potential interfering species in the serum samples.

Eventually, quality parameters such as the stability, repeatability and reproducibility of the proposed modified IJP Au electrode were evaluated to ensure the robustness and durability of the electrochemical sensor (Table S2). Repeatability shows how accurate some consecutive measurements are. For this reason, this parameter was studied by performing three successive calibrations with the same sensor in a period of one day. The sensitivities obtained were compared, and the relative standard deviation (RSD %) was calculated. The average sensitivity obtained for this electrode was 14 ± 2 nA/nM and RSD was 14% for β–CD-S/Au–NPs@rGO, and a sensitivity of 5 ± 2 nA/nM and RSD of 38% for γ–CD-S/Au–NPs@rGO were obtained. It is clear that the β–CD-S/Au–NPs@rGO sensor not only has a higher sensitivity but is also more consistent with measurements than the γ–CD-S/Au–NPs@rGO sensor. As these sensors were aimed at point-of-care applications, a 14% RSD was deemed acceptable. However, the 38% variation between signals for the γ–CD- sensor is too big of a change for even single-use devices. Reproducibility is studied to determine how comparable a set of sensors from the same fabrication batch, which were modified under the same experimental conditions, are. Thus, three different β–CD-S/Au–NPs@rGO and γ–CD-S/Au–NPs@rGO microsensors were prepared and evaluated to compare their amperometric current responses. For this aim, 10 nM of T4 was evaluated using three different sensors. The resulting RSD was 0.7% for β–CD and 4.2% for γ–CD, confirming that the preparation method is highly reproducible.

Short-term stability establishes the lifetime of a sensor in successive measurements. To investigate this parameter, successive amperometric measurements of 10 nM T4 solution were made at the same sensor (β–CD and γ–CD), undergoing deep-cleaning after each measurement with a PBS solution that contains an ionic background of 0.1 M KCl. Successive amperometric experiments (n = 10) were carried out under the same experimental conditions, obtaining an intensity average value of 223 ± 4 nA for β–CD and 42 ± 1 nA for γ–CD, where RSD was 1.8% and 2.5%, respectively. Long-term stability establishes the lifetime of the sensor. Hence, this parameter was studied using multiple calibration curves (n = 3) and performed with the same sensor on alternative days for a month. The sensors were kept dry and out of light for one month. Sensitivities were compared, and it was concluded that no significant differences in sensitivities were obtained (t_cal_ = 1.226 < t_tabulated_ = 2.120 for β–CD and t_cal_ = 1.053 < t_tabulated_ = 2.120 for γ–CD with a 95% confidence level). These results allow us to conclude that the amperometric sensors can repeat the same behavior during an evaluated period, confirming the proper stability of the sensors during a given period and their high robustness of the developed biosensors.

## 4. Conclusions

In this work, an Au microelectrode amended with an entrapment agent for sensing T4 is presented. Different approaches using two kinds of cyclodextrin were studied. We have synthesized a graphene-based hybrid-nanomaterial (x–CD-S/Au–NP@rGO) for the development of a sensitive T4 amperometric sensor by the incorporation of β–CD-SH and γ–CD-SH as preconcentration agents into the graphene-based electronic transducer via supramolecular chemistry. The hybrid-nanomaterial was deposited using the drop-casting technique over Au microelectrodes. In this work, it has been demonstrated that nanostructured carbon materials modified with Au–NPs could be promising materials for the enhancement of the electron-transfer capabilities of conductive materials. In addition, a consistent support for attaching thiolated molecules, which may be used as preconcentration agents, has also been demonstrated. The strength of the gold–thiol interactions provided the basis for the fabrication of a robust 2D hybrid nanomaterial that contains the carrying elements.

Both the β–CD-SH and γ–CD-SH hybrid nanomaterials showed an optimal receptor–ligand interaction with T4. However, the best electroanalytical properties were obtained for the β–CD-S-based nanomaterial. For β–CD-S, an enhanced sensitivity, limit of detection (LOD) and limit of quantification (LOQ) measurements were achieved compared to γ–CD-S. Additionally, an equivalent linear response range for β–CD-S and γ–CD-S was obtained. Further, differences in sensitivity can be attributed to a more effective supramolecular receptor–ligand interaction which is afforded by β–CD relative to γ–CD. The β–CD-S/Au–NP@rGO sensor presents the lowest LOD compared to the other electrochemical carbon-based sensors found in the literature.

The electroanalytical features of the proposed modified IJP Au microelectrode combined with its tunable sensitivity based on rGO hybrid nanomaterials opens a wide range of applications for sensing purposes, such as clinical, pharmacological and biomedical investigations. This approach presents a promising alternative for the detection of thyroid hormones and addresses some of the limitations associated with traditional methods.

## Figures and Tables

**Table 1:** Quality parameters of the proposed microsensors: LOD, LOQ and linear range.

Modification	Sensitivity (nA/nM)	Intercept	R2 (n = 9)	LOD (nM)	LOQ (nM)	Linear Range (nM)
β–CD-S/Au–NPs@rGO	14.7 ± 0.4	30.2 ± 1.2	0.994	0.25	0.8	0.8–6
γ–CD-S/Au–NPs@rGO	6.6 ± 0.1	8.4 ± 0.4	0.997	0.89	1.8	1.8–6

## Data Availability

The data presented in this study are available upon request from the corresponding author. The data are not publicly available because the repository that is used to keep the data is a private one provided by the university.

## Supplementary Materials

The following supporting information can be downloaded at: https://www.mdpi.com/article/10.3390/nano14050403/s1, Figure S1: Regulation of thyroid hormones. Activation of the pituitary gland by TRH hormone for the release of TSH (A). Activation of the thyroid gland by TSH for the release of T3 and T4 (B). Propagation of hormones to organs (C). Different diseases caused according to the concentration of T4 and TSH hormones in the blood (D). Figure S2: (A) Stability test for different studied rGO dispersions (N,N-Dimethylformamide (DMF), chitosan, lignin, polyethylene glicol (PEG) and polylactic acid (PLA)) to form the composite by immersing the modified electrodes in water. (B) Cyclic voltammetry response of two electrodes modified with two of the most suitable mixtures to form the composites. Experimental conditions: [Fe(CN)_6_]^3−/4−^ 0.01 M in KCl 0.1 M at scan rate: 10 mV·s^−1^. Figure S3: Morphological characterization of nanomaterials. HR-TEM images of (A) rGO, (B) Au–NPs@rGO, (C) β–CD-S/Au–NPs@rGO and (D) γ–CD-S/Au–NPs@rGO. (E) Histogram corresponding to size distribution of NPs. Figure S4: EDS spectra of (A) Au–NPs@rGO, (B) β–CD-S/Au–NPs@rGO, (C) γ–CD-S/Au–NPs@rGO. Figure S5: TGA diagram corresponding to Au–NPs@rGO (black), β–CD-S/Au–NPs@rGO (orange) and γ–CD-S/Au–NPs@rGO (blue). Figure S6: Electrochemical behavior of the different graphene–based nanocomposite in a 0.1 M KCl containing 0.01 M [Fe(CN)_6_]^3−/4−^. Experiments were carried out by CV at scan rate: 10 mV·s^−1^. Figure S7: CV depicts the influence of scan rate from 10 mV·s^−1^ to 100 mV·s^−1^ for 1 μM of T4 sensing in a 0.1 M NaOH 1:9 in EtOH solution on PBS with 0.1 M KCl at the (A) β–CD-S/Au–NPs@rGO / (B) γ–CD-S/Au–NPs@rGO sensors. Figure S8: Chronoamperometry measurements at different T4 concentrations for (A) β–CD-S/Au–NPs@rGO/(B) γ–CD-S/Au–NPs@rGO sensors. Experimental conditions: 0.1 M NaOH 1:9 in EtOH solution on PBS with 0.1 M KCl and Ew: +0.75 V vs Ag/AgCl. Figure S9: Calibration curves obtained, from β-cyclodextrin, from the representation of I_p_^a^ vs. [T4] for n = 3 was carried out by chronoamperometry in a 0.1 M NaOH 1:9 in EtOH solution on PBS with 0.1 M KCl. Ew: +0.75 V vs. Ag/AgCl. Table S1: Electrochemical characterization values obtained from CV. Table S2: Analytical data extracted for the repeatability (calibrations), reproducibility (measurements), and stability (electrodes) statistics.
