# Supplementary material for: Amperometric Inkjet-Printed Thyroxine Sensor Based on Customized Graphene and Tunned Cyclodextrins as the Preconcentration Element
Source: Nanomaterials (Basel). 2024 Feb 22;14(5):403. doi: 10.3390/nano14050403 (PMC10934536; doi:10.3390/nano14050403)
Supplement: Supplementary file 1 [file nanomaterials-14-00403-s001.zip › nanomaterials-2827007-supplementary.pdf]

*nanomaterials*

*Supplementary Materials*

# Amperometric Inkjet-Printed Thyroxine Sensor Based on Customized Graphene and Tunned Cyclodextrins as the Preconcentration Element

**María Jesús Ortiz-Aguayo [1], Franc Paré [1,2], Gemma Gabriel [3,4] and Mireia Baeza [1,2,*]**

[1] Department of Chemistry, Faculty of Science, Edifici C-Nord, Universitat Autònoma de Barcelona, Carrer dels Tillers, 08193 Bellaterra, Spain; mortiz@icmab.es (M.J.O.-A.); franc.pare@uab.cat (F.P.)
[2] GENOCOV Research Group, Universitat Autònoma de Barcelona, 08193 Bellaterra, Spain
[3] Instituto de Microelectrónica de Barcelona, IMB-CNM (CSIC), Esfera UAB, Campus Universitat Autònoma de Barcelona, 08193 Bellaterra, Spain; gemma.gabriel@imb-cnm.csic.es
[4] Centro de Investigación Biomédica en Red de Bioingeniería, Biomateriales y Nanomedicina (CIBER-BBN), 28029 Madrid, Spain
* Correspondence: mariadelmar.baeza@uab.cat

### *Graphene materials synthesis*

The graphene-based materials are synthesized with a modified Hummers' method. It's divided into two main steps. Starting from graphite, graphene oxide (GO) is generated with the aim of breaking the different carbon layers. Followingly, GO is reduced in the presence of a metal salt to both generate reduced graphene oxide (rGO) and the metallic nanoparticles.

To oxidize the graphite, in a 500 mL round flask 5 g of laminar graphite, 2.5 g on $NaNO_3$ and 115 mL of 97% $H_2SO_4$ are introduced, ant the flask placed in an ice bath. Then, 15 g of $KMnO_4$ are slowly added whilst stirring. Once finished adding the $KMnO_4$ the solution is heated to 45 ºC for 30 minutes. It is later cooled down to room temperature, 230 mL of ultrapure water are incorporated and left to stir for 15 more minutes. The resulting solution is transferred to a larger container with 700 mL of additional ultrapure water and 17.5 mL of $H_2O_2$ to transform into $Mn^{2+}$ the permanganate leftovers. Followingly, the solution is spread into different tubes and centrifuged in 10% HCl until the subproducts can be extracted; then the process is repeated with ultrapure water until reaching a neutral pH. The final step is taking the solid product into an oven at 80 ºC for 24 h to dry off.

To reduce the GO 500 mg of the obtained dry powder are added in 500 mL of ultrapure water and sonicated for 1h to disperse the graphene layers. Afterwards, the solution is heated to 95 ºC in a silicone bath and brought to a slightly alkaline pH (9-10) using 25% $NH_3$. Next, ascorbic acid 2 mM is added gradually which, together with the alkaline pH, reduces a high number of carboxylic groups. For the formation of gold nanoparticles on the rGO (AuNPs@rGO), 350 mg of GO are dispersed in 250 mL of ultrapure water with 394 mg of $HAuCl_4$. Instead of ascorbic acid, the reducing agent is replaced by 250 mL of 0.1 M $NaBH_4$. The stronger reducing agent attacks the $AuCl_4^-$ ions to form Au nanoparticles, mainly over the non-reduced carboxylic groups on the rGO, which act as catalytic points. After the addition of either reducing agent, the solutions are left to continue stirring for 1 h and then centrifuged at 3500 rpm in ultrapure water until neutral pH is reached. The resulting powder is dried in an oven at 80 ºC for 24 h.

### *Content of β-CD-S and γ–CD-S in hybrid nanomaterial*

In addition, quantification of Au content was carried out by TGA analysis. Regarding **Figure S5**, an amount of 52.9 wt% of Au is determined. Moreover, the number of entrapment agent (β-CD-SH and γ–CD-SH) deposited on the rGO sheets was obtained following the supporting information from reference [27]:

The weight of Au (WAu) formed experimentally from TGA is: $W_{Au}$= 52.9 g Au. The WNPs for Au–NPs of 4.40 nm is: WNPs= $0.71 \cdot 10^{-18}$ g Au–NPs. From that, it was calculated the weight of each nanoparticle (WNPs). From this, the number of Au–NPs is: NNPs= WAu/WNPs. Accordingly, the number of Au–NPs is: NNPs= $7.5 \cdot 10^{19}$. The number of biorecognition agent (Nβ-CD-S) is: 24.8 g β–CD-S (from TGA), which correspond to Nβ-CD-S = 24.8 g β–CD-S· $MW^{-1}$ · NAv = $1.20 \cdot 10^{22}$ β–CD-S molecules. Finally, the number of biorecognition agent per nanoparticles is: n β–CD-S = Nβ-CD-S / NAu–NPs and thus, nβ–CD-S ≈ 160. For γ–CD-S, the number of biorecognition agent (Nγ-CD-S) is: 17.9 g γ–CD-S (from TGA), which correspond to Nγ-CD-S = 17.9 g γ–CD-S · $MW^{-1}$ · NAv = $6.94 \cdot 10^{21}$ γ–CD-S molecules. Finally, the n γCD-S= Nγ-CD-S / NAu–NPs and thus, nγ–CD-S ≈ 92.

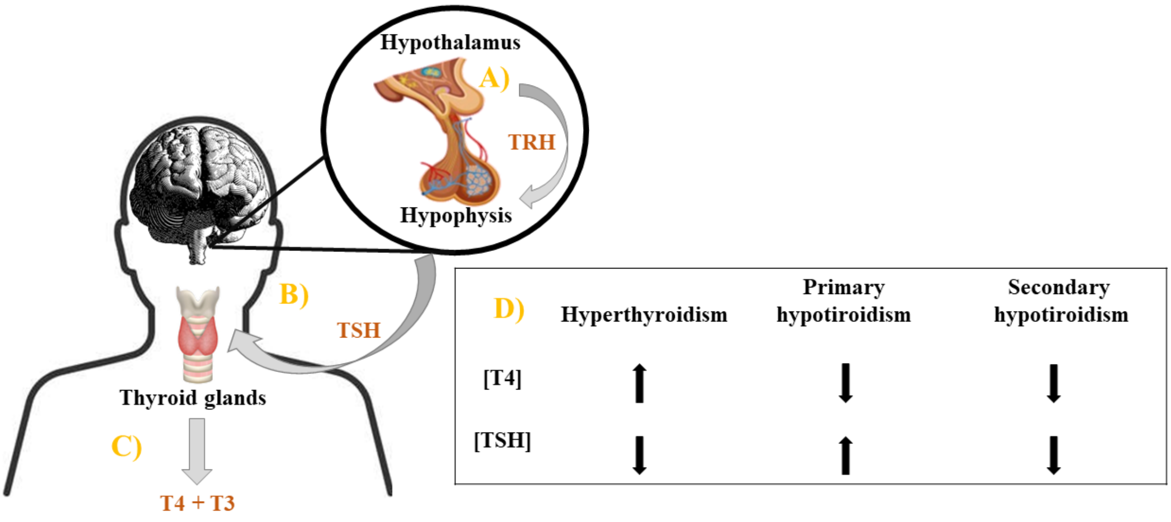


**Figure S1.** Regulation of thyroid hormones. Activation of the pituitary gland by TRH hormone for the release of TSH (A). Activation of the thyroid gland by TSH for the release of T3 and T4 (B). Propagation of hormones to organs (C). Different diseases caused according to the concentration of T4 and TSH hormones in the blood (D).

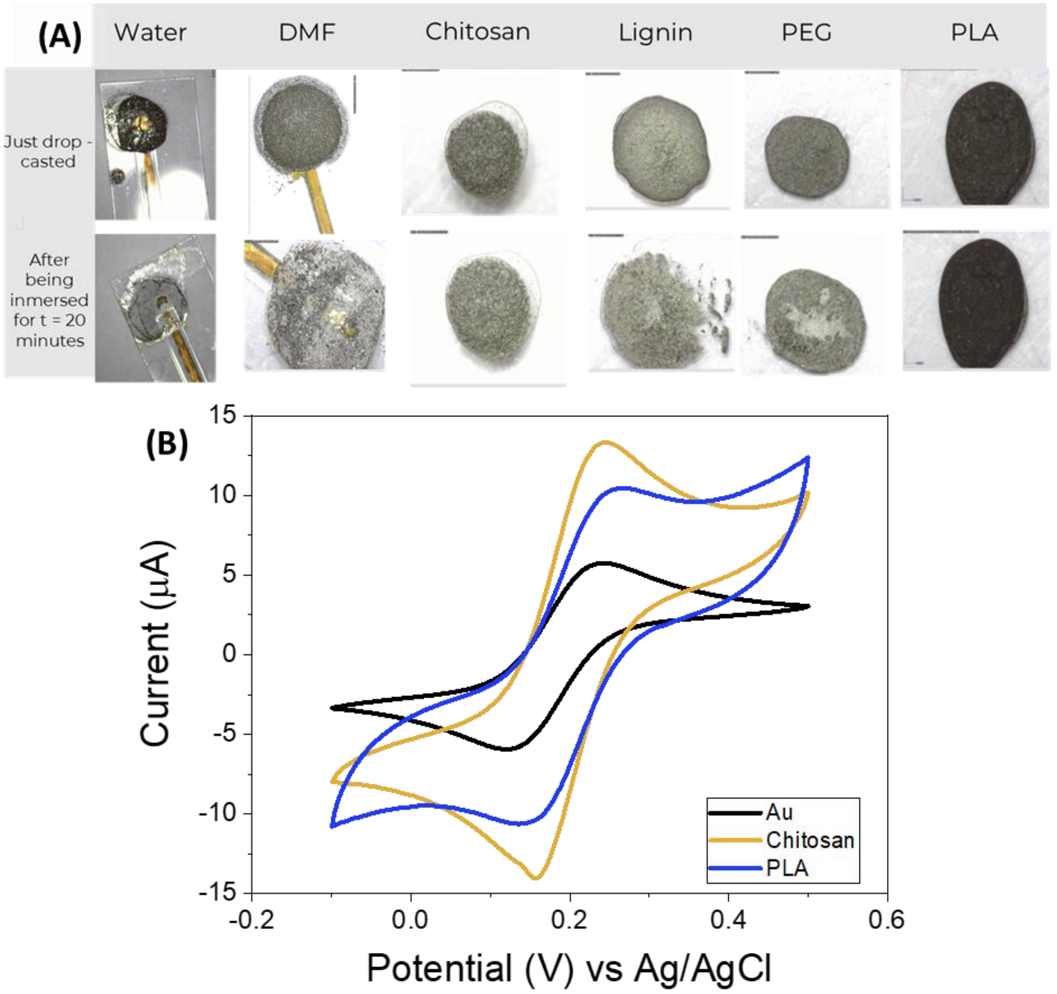


**Figure S2.** (A) Stability test for different studied rGO dispersions (N,N- Dimethylformamide (DMF), chitosan, lignin, polyethylene glicol (PEG) and polylactic acid (PLA)) to form the composite by immersing the modified electrodes in water. (B) Cyclic voltammetry response of two electrodes modified with two of the most suitable mixtures to form the composites. Experimental conditions: $[Fe(CN)_6]^{3-/4-}$ 10 mM in KCl 0.1 M at 10 $mV{\cdot}s^{-1}$ scan rate.

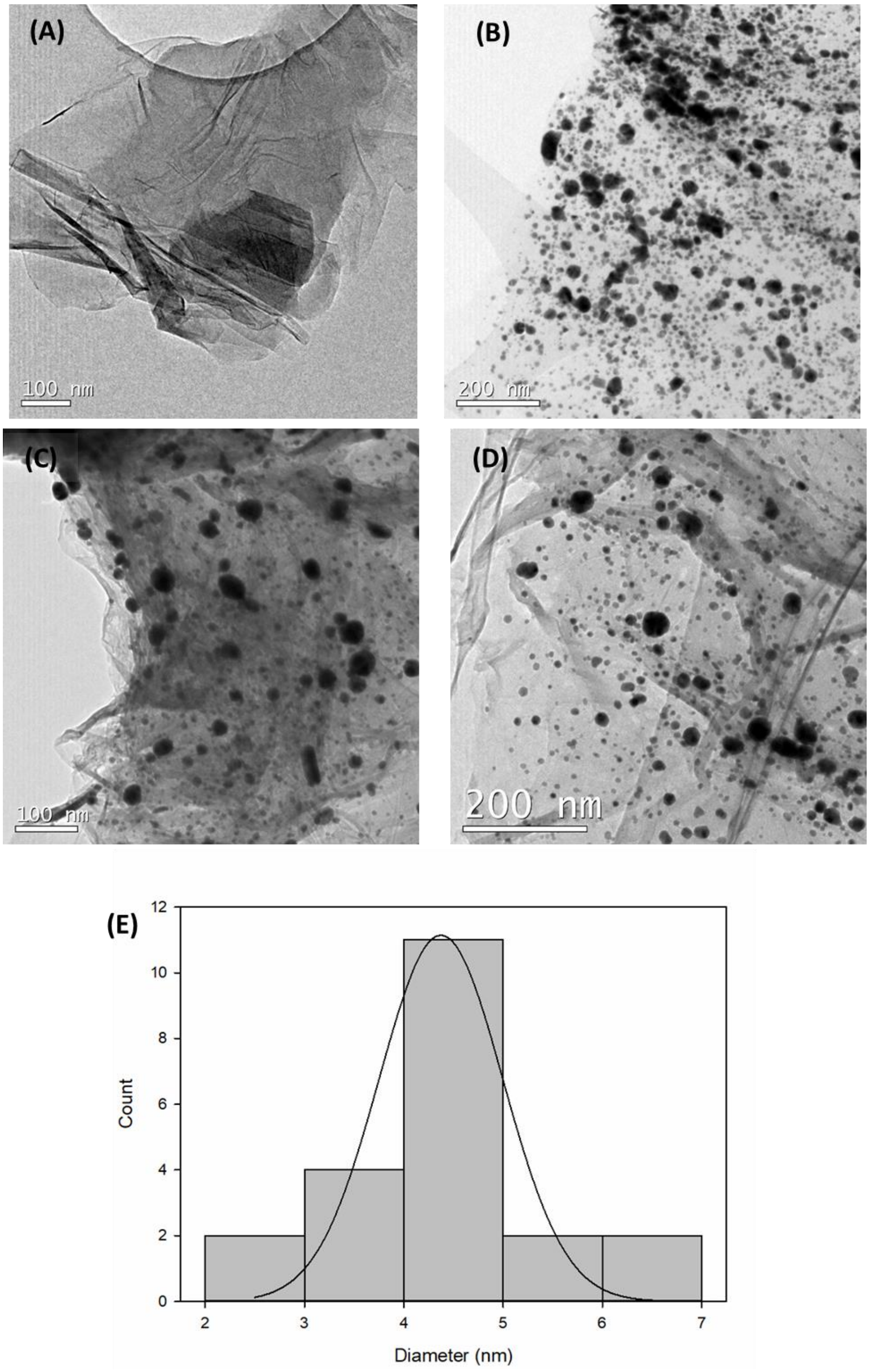


**Figure S3.** Morphological characterization of nanomaterials. HR-TEM images of (A) rGO, (B) Au-NPs@rGO, (C) β–CD-S/Au–NPs@rGO and (D) γ–CD-S/Au–NPs@rGO. (E) Histogram corresponding to size distribution of Au-NPs.

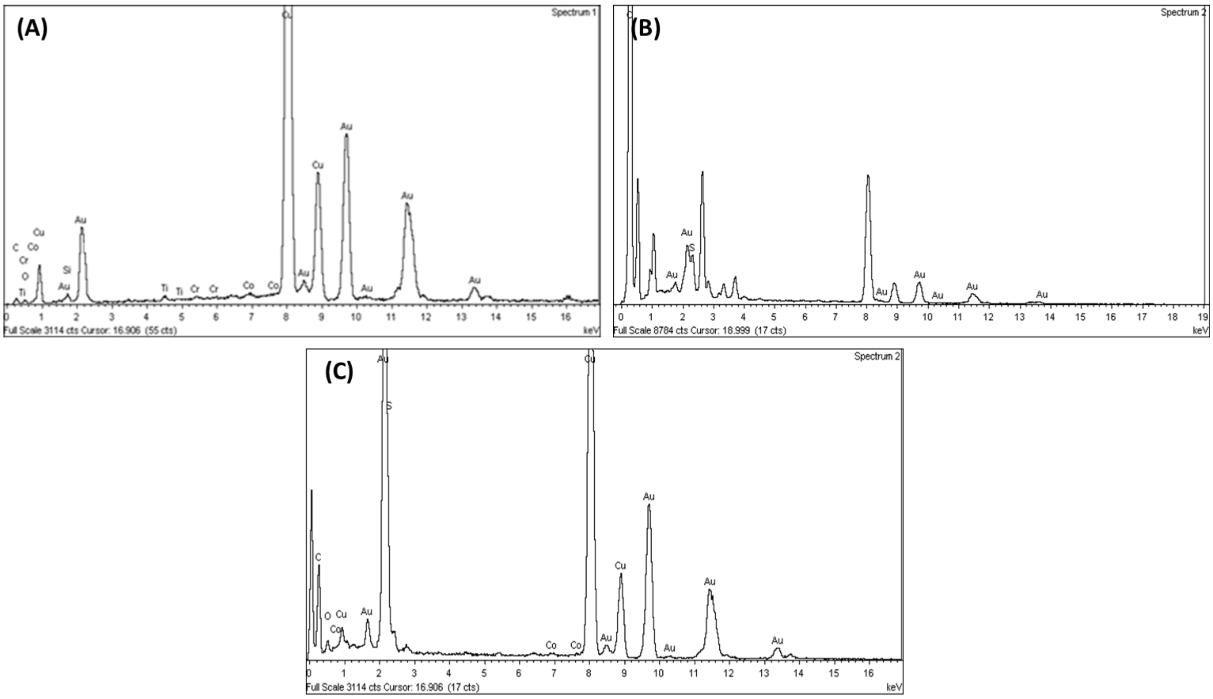


**Figure S4.** EDS spectra of (A) Au-NPs@rGO, (B) β–CD-S/Au–NPs@rGO and (C) γ–CD-S/Au–NPs@rGO.

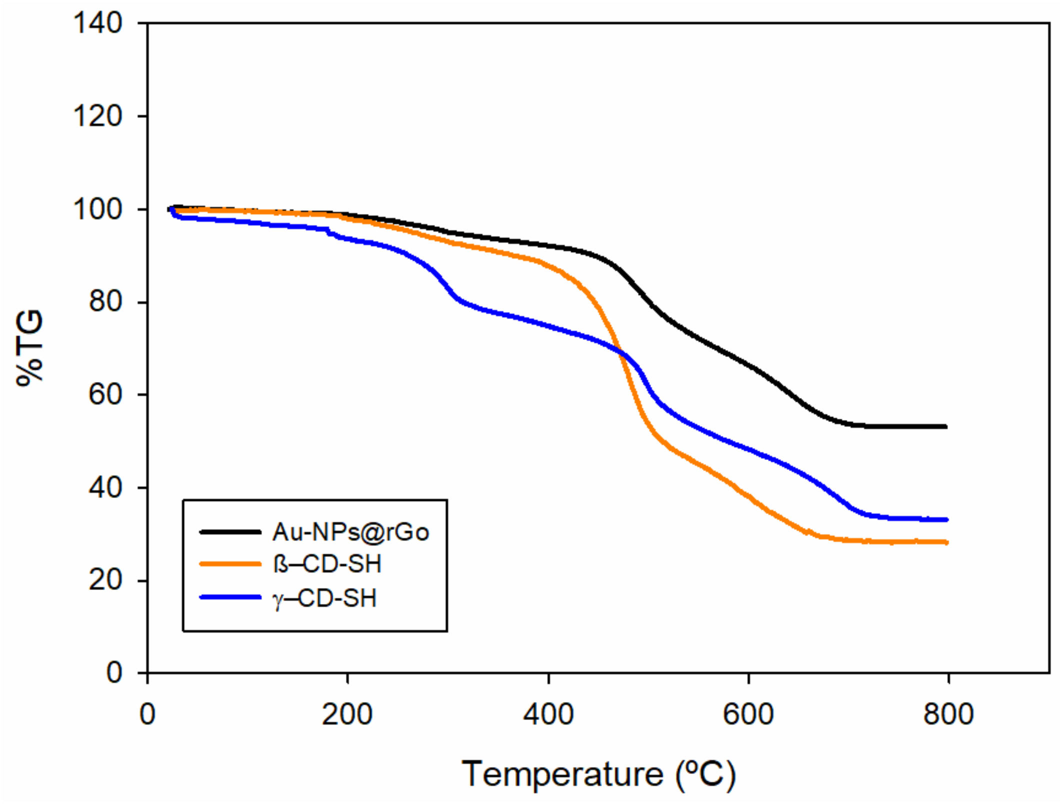


**Figure S5.** TGA diagram corresponding to Au-NPs@rGO (black), β–CD-S/Au–NPs@rGO (orange) and γ–CD-S/Au–NPs@rGO (blue).

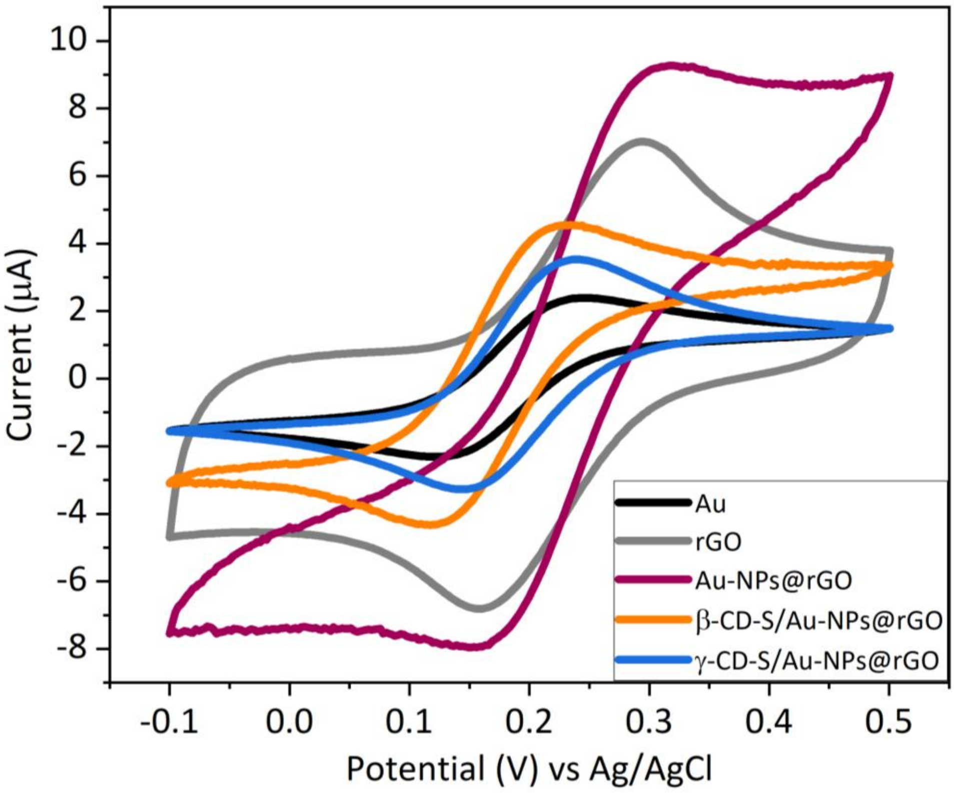


**Figure S6.** Electrochemical behavior of the different graphene–based nanocomposite electrodes in a 0.1 M KCl containing 10 mM $[Fe(CN)_6]^{3-/4-}$. Experiments were carried out by CV at scan rate: 10 mV·s$^{-1}$.

**Table S1.** Electrochemical characterization values obtained from CV.

| Electrode modification | ΔE (V) | $I_0$ (μA) | $R_{ct}$ (Ω) | $I_p^c$ (μA) | A (mm²) |
|---|---|---|---|---|---|
| **bare Au** | 0.095 | 2.40 | 10532 | -2.57 | 2.96 |
| **rGO** | 0.112 | 0.19 | 693 | -6.77 | 44.1 |
| **Au-NPs@rGO** | 0.102 | 1.12 | 3994 | -7.93 | 7.55 |
| **β-CD-S/Au-NPs@rGO** | 0.186 | 5.53 | 7147 | -3.28 | 5.33 |
| **γ -CD-S/Au-NPs@rGO** | 0.085 | 4.19 | 6007 | -4.08 | 3.55 |

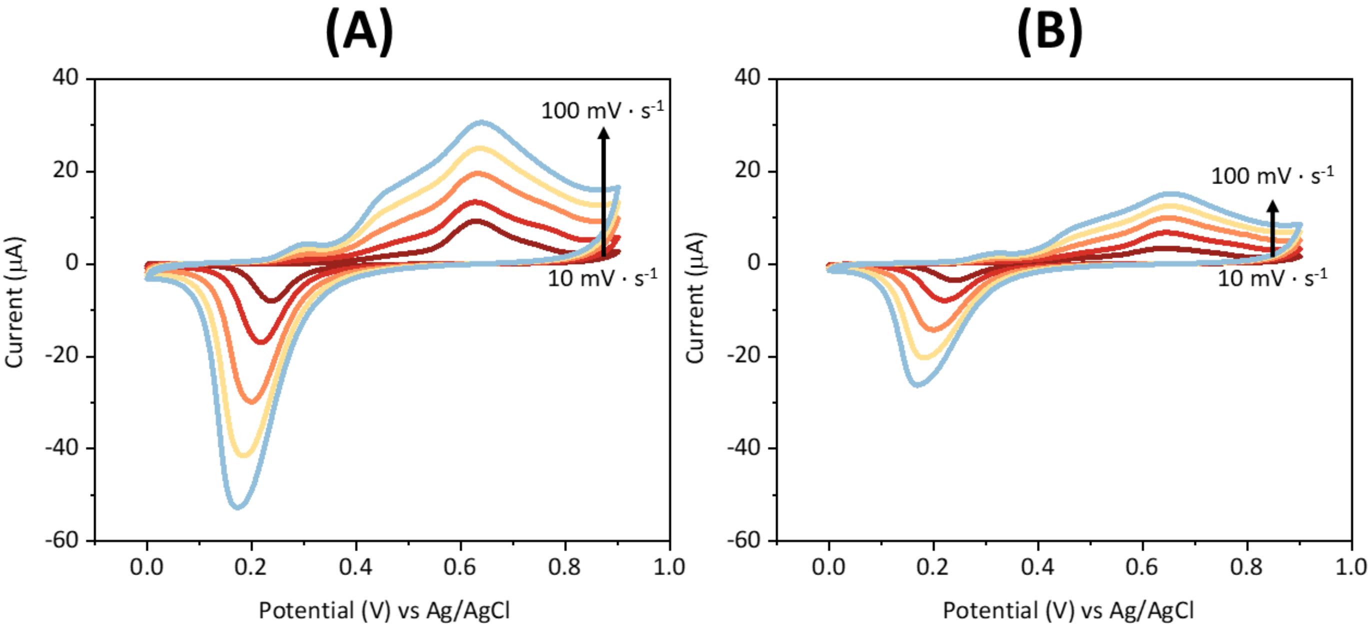


**Figure S7.** CV depicts the influence of scan rate of 10, 25, 50, 75 and 100 mV·s$^{-1}$ for 1 µM of T4 sensing in a 0.1 M NaOH 1:9 in EtOH solution on PBS with 0.1 M KCl at the (A) β–CD-S/Au–NPs@rGO / (B) γ–CD-S/Au–NPs@rGO sensors.

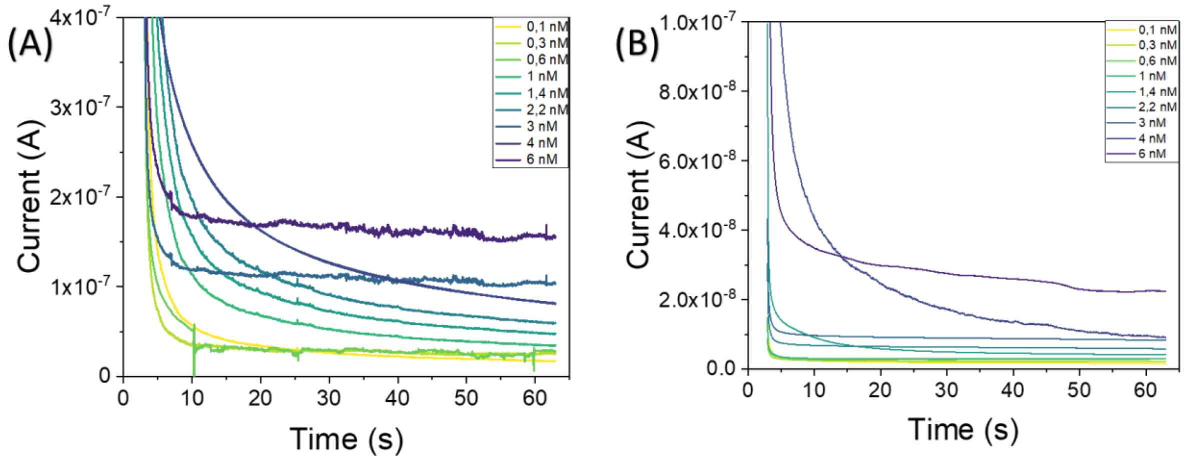


**Figure S8.** Chronoamperometry measurements at different T4 concentrations for (A) β–CD-S/Au–NPs@rGO / (B) γ–CD-S/Au–NPs@rGO sensors. Experimental conditions: 0.1 M NaOH 1:9 in EtOH solution on PBS with 0.1 M KCl and Ew: +0.75 V vs Ag/AgCl.

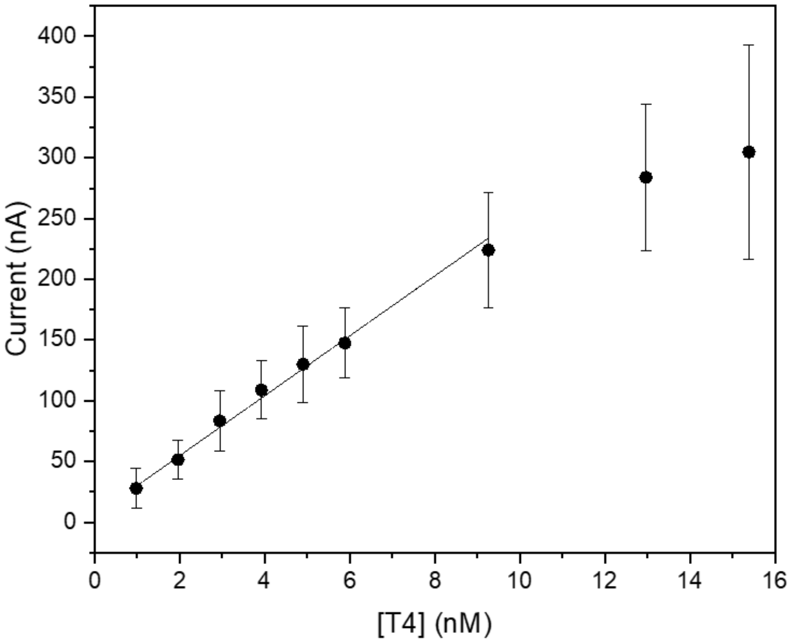


**Figure S9.** Calibration curves obtained, from β-CD-S/Au–NPs@rGO sensor, from the representation of $I_p{}^a$ vs. [T4] for n=3 was carried out by chronoamperometry in a 0.1 M NaOH 1:9 in EtOH solution on PBS with 0.1 M KCl. Ew: +0.75 V vs Ag/AgCl.

**Table S2.** Analytical data extracted for the repeatability (calibrations), reproducibility (measurements), and stability (electrodes) statistics.

| *Study* | *Step* | *[T4] (nM)* | *Signal (mA)* | *Sensitivity (nA/nM)* | *RSD (%)* |
|---|---|---|---|---|---|
| *β-CD-S/Au-NPs@rGO* | | | | | |
| **Repeatability** | n = 3 | 0.3-6 | | 14 ± 2 | 14 |
| | 1 | | 223 | | |
| **Reproducibility** | 2 | 10 | 222 | | 0.7 |
| | 3 | | 225 | | |
| **Long term stability** | 1 | | | 14.7 ± 0.4 | |
| | 2 | 0.3-6 | | 13.1 ± 0.6 | |
| | 3 | | | 12.9 ± 0.6 | |
| | 1 | | 220 | | |
| **Short term stability** | 2 | | 227 | | |
| | 3 | | 217 | | |
| | 4 | | 218 | | |
| | 5 | 10 | 228 | | 1.8 |
| | 6 | | 220 | | |
| | 7 | | 222 | | |
| | 8 | | 224 | | |
| | 9 | | 226 | | |
| | 10 | | 223 | | |

| | | | | | |
|---|---|---|---|---|---|
| *γ -CD-S/Au-NPs@rGO* | | | | | |
| **Repeatability** | n = 3 | 1.2-6 | | 5 ± 2 | 38 |
| | 1 | | 37.2 | | |
| **Reproducibility** | 2 | 10 | 40.5 | | 4.2 |
| | 3 | | 38.9 | | |
| **Long term stability** | 1 | | | 6.8 ± 0.1 | |
| | 2 | 1.2-6 | | 6.6 ± 0.1 | |
| | 3 | | | 4.7 ± 0.5 | |
| | 1 | | 42 | | |
| **Short term stability** | 2 | | 43 | | |
| | 3 | | 42 | | |
| | 4 | | 44 | | |
| | 5 | 10 | 43 | | 2.5 |
| | 6 | | 41 | | |
| | 7 | | 41 | | |
| | 8 | | 43 | | |
| | 9 | | 41 | | |
| | 10 | | 42 | | |

**Disclaimer/Publisher's Note:** The statements, opinions and data contained in all publications are solely those of the individual author(s) and contributor(s) and not of MDPI and/or the editor(s). MDPI and/or the editor(s) disclaim responsibility for any injury to people or property resulting from any ideas, methods, instructions or products referred to in the content.
